# Attentional Distraction during Exercise in Overweight and Normal-Weight Boys

**DOI:** 10.3390/ijerph120303077

**Published:** 2015-03-13

**Authors:** Benedicte Deforche, Ilse De Bourdeaudhuij

**Affiliations:** 1 Department of Public Health, Ghent University, De Pintelaan 185, 9000 Gent, Belgium; 2 Department of Human Biometrics and Biomechanics, Vrije Universiteit Brussel, Pleinlaan 2, 1050 Brussel, Belgium; 3 Department of Movement and Sports Sciences, Ghent University, Watersportlaan 2, 9000 Gent, Belgium; E-Mail: Ilse.Debourdeaudhuij@UGent.be

**Keywords:** adolescents, music, obesity, physical activity, running performance

## Abstract

The purpose of this study was to investigate the effect of attentional distraction on field running distance and activity intensity during an exercise session in normal-weight and overweight youngsters and to investigate potential mediators. Fifty-three 12–14 yr-old boys participated twice in a 12-min running test and a 20-min exercise session, once with attentional distraction (by listerning to music) and once without distraction (counterbalanced randomised controlled design). At the end of the endurance test running distance was recorded. During the exercise session activity intensity was assessed by accelerometers. After each experiment, rate of perceived exertion (RPE) was estimated and seven questions were asked about how participants experienced the experiment. Both overweight and normal-weight boys ran further during the running test with music (*p* < 0.05) and this effect was mediated by a decrease in feelings of annoyance. During the exercise session with music, both overweight and normal-weight boys exercised less at low and high intensity and more at moderate and very high intensity (*p* < 0.01) and this effect was mediated by a decrease in RPE. We can conclude that attentional distraction has a positive effect on running distance on a field endurance test and on activity intensity during an exercise session through different mechanisms in both overweight and normal-weight boys.

## 1. Introduction

Overweight youngsters are found to be less active or to be active at a lower intensity than normal-weight counterparts [1,2]. As regular physical activity of high enough intensity is essential in the prevention of obesity [3,4], efforts should be made to increase physical activity adherence in overweight youngsters. 

Enjoyment is the most important predictor of physical activity in children [5,6,7]. Unfortunately, overweight youngsters generally do not choose to be active because they like it, but rather because they hope to lose weight or look better [8]. These extrinsic motives may not encourage continued participation in physical activity, since weight-loss directly attributable to increased physical activity is usually small [9]. The lack of direct effects of physical activity may disappoint overweight youngsters and cause drop-out. In addition, overweight youngsters perceive more barriers towards physical activity [8,10,11]. They find it more exhausting and report more physical complaints such as side stiches, knee pain, suffocating feeling, excessive sweating, *etc.* Moreover, a higher perception of barriers in overweight youngsters is related to a lower participation in physical activities [11]. Since physical activity plays an important role in the prevention of overweight, it is important to find solutions to overcome these barriers and to intrinsically motivate overweight youngsters to be physically active. A key element is that overweight youngsters should perform enough activity to substantially increase total energy expenditure. This can be accomplished either by prolonging the duration of the activity or by raising its intensity. Generally, the importance of exercise duration, rather than intensity, is emphasized to promote a significant fat oxidation and to prevent drop-out [12]. The intensity of physical activity has been found to be negatively associated with exercise adherence in overweight children and adults [13,14]. This could be due to the fact that higher intensity activities entail more physical complaints and are therefore experienced as less pleasant. However, especially activities of moderate to high intensity have shown to contribute to the prevention of weight gain in adults [15,16]. It might be interesting to investigate ways to increase exercise intensity in overweight youngsters without increasing physical complaints or annoyance. 

It is possible that attentional distraction may increase adherence to higher intensity activities in overweight youngsters. Previous experiments in athletes showed that by focusing attention to external stimuli (such as music) instead of internal sensory information (such as heart rate, breathing, bodily symptoms) running performances increased [17]. Although the effect of attentional distraction is well established in athletes, only one study has investigated this issue in overweight people. A previous experiment in obese children and adolescents [18] showed that attentional distraction by music has a positive effect on perseverance during a treadmill test. This study was performed in a controlled laboratory environment. As findings from a laboratory setting might not be transferable to field settings and the effect of attentional distraction on activity intensity during an exercise session has not been studied yet, further research is needed to investigate whether attentional distraction is also useful to increase performance in field conditions and during exercise programs in overweight youngsters. 

The purpose of this study is to investigate the effect of attentional distraction in overweight *versus* normal-weight youngsters on: (1) running distance in the field and on intensity of activity during an exercise session (primary outcomes) and (2) rate of perceived exertion (RPE), degree of annoyance, attention given to bodily sensations or thoughts about being able to carry on (secondary outcomes). We hypothesize that attentional distraction will have a positive effect on primary and secondary outcomes in both exercise conditions. As overweight youngsters report more physical complaints while exercising [8], we further hypothesize that this effect will be stronger in overweight compared to normal-weight youngsters. In addition, we want to investigate whether the effect of attentional distraction on exercise performance is mediated by changes in rate of perceived exertion (RPE), degree of annoyance, attention given to bodily sensations or thoughts about being able to carry on.

## 2. Methods

### 2.1. Participants

Four classes of 12 to 14 year old boys (N = 53) were recruited in a boys’ technical/vocational school with high overweight prevalence and were grouped into normal-weight or overweight according to international cutoffs for overweight in children [19]. Based on differences in running performance with and without distraction found in a previous laboratory study in obese children and adolescents [18] an *a priori* power analysis was conducted. This analysis showed that to study 2 × 2 within-between interactions with a power of 0.80 (given a 0.05 level of significance), a total sample size of minimum 38 subjects was needed. All participants were orally informed about the purpose of this study and received an information letter for their parents. Written informed consent was obtained from all boys and their parents before participating in the study. The study was conducted in accordance with the Declaration of Helsinki, and the protocol was approved by the ethical committee of the Ghent University Hospital. 

### 2.2. Procedure

Participants performed twice a field running test and participated twice in an exercise session, once with attentional distraction and once without distraction. To control for order effects, in both the running test and the exercise session, half of the classes started with distraction and half of the classes without distraction (counterbalanced design). As randomization of order of the conditions was at the class level and not individual level, this was a quasi-experimental randomized controlled design. Since tests were performed during physical education classes, they took place each time at the same day of the week and the same time of the day. There were two weeks between each test. No encouragement was given during the tests. 

### 2.3. Measurements

#### 2.3.1. Anthropometric Measurements

Height was measured to the nearest 0.1 cm using a stadiometer (Holtain Ltd, Crymmych, Pembs, UK). Body mass was measured to the nearest 0.1 kg on a digital balance scale (Seca, max 200 kg, Hamburg, Germany) with the participant wearing lightweight clothing and no shoes. BMI was calculated from height and weight measures (weight in kg/height in m^2^).

#### 2.3.2. Physical Activity

Physical activity was estimated using a modified version of the Baecke Questionnaire [20], previously used to assess physical activity in 12–18 year old youngsters [8,21]. Responses to 13 questions were scored on a 5-point scale and resulted in two indices reflecting physical activity during sport (sport index) and during leisure time excluding sport (leisure time index). Items regarding physical activity during work were omitted for this study. A sport score was calculated from a combination of the intensity of the (organised or non-organised) sport which was played, the amount of time per week playing that sport, and the proportion of the year in which the sport was played regularly. The sport index was calculated from the sport score, level of activity in comparison with friends and frequency of sweating during leisure time physical activities. The leisure time index was based on the amount of television watching and the frequency and daily amount of walking or cycling as a means of transportation. The validity of the Baecke Questionnaire for the assessment of physical activity has been previously reported [22,23]. 

#### 2.3.3. Field Running Performance

Field running performance was assessed by the Cooper Test [24] which is a 12 min running test. Different investigators found high correlations (0.82 < r < 0.94) between VO_2max_ and performance on the 12 min running test in young adults [25,26]. The tests were performed on an outdoor athletics track. Weather conditions were similar on the test days with and without attentional distraction. In the condition with attentional distraction, participants were wearing a portable audio player (Sony D-EJ 750, G-protection). As the largest benefits to RPE were found with music that is preferred [27], each participant could bring his own favourite music. Music volume was standardised, however tempo was not controlled. At the end of the endurance tests running distance was recorded.

#### 2.3.4. Activity during Exercise Session

The exercise session consisted of a 20 min exercise circuit with focus on movements with vertical displacement of the body. Participants were not wearing a portable audio player, but music was played on a CD-player (AZ 2030, Philips, Eindhoven, The Netherlands). It was a mix of popular hits with a fast tempo and a strong rhythm. During the exercise sessions physical activity was assessed by accelerometry (model 7164, Computer Science Application, Inc., Shalimar, FL, USA). The accelerometers were set to measure activity counts in an epoch time of one minute. Activity counts are the summation of the accelerations measured over the epoch and are used to determine the intensities of activities performed. A distinction was made between minutes of less than 1952 counts (less than 3 METS), 1952 to 5724 counts (3.0 to 5.99 METS), 5725 to 9498 counts (6.0 to 8.99 METS) and more than 9498 counts (more than 8.99 METS), corresponding respectively to activity of light, moderate, high and very high intensity [28]. Participating boys were imposed to wear the accelerometer above the right hipbone, underneath the clothes. Accelerometers were held in place with an elastic belt and adjustable buckle. The accelerometer has been shown to be a valid and reliable tool for the assessment of physical activity in children [29,30,31]. 

#### 2.3.5. Rate of Perceived Exertion

After each test or exercise session, rate of perceived exertion (RPE) was obtained using the Borg 15-point category scale [32]. RPE is defined as the subjective intensity of effort, strain, discomfort and/or fatigue experienced during exercise. The Borg 15-point category scale consists of numbered categories, 6–20, and verbal cues from “very, very light” to “very, very hard”. This scale is commonly used to measure RPE during exercise in normal-weight and overweight youth [33,34,35].

#### 2.3.6. Questionnaire

After each test or exercise session seven questions were asked about how the participants experienced the test or the session using a 5-point scale (1 = not at all, 5 = very much). Participants reported (1) how annoying they experienced the test (or exercise session), (2) how much attention was given to bodily sensations during the test (or exercise session), (3) how often they had thoughts about being able to carry on with the test (or exercise session), (4) to what extent they liked the music, (5) to what extent they could listen to the music during the test (or exercise session), (6) how pleasant they found the test (or exercise session) while listening to music, and (7) to what extent they believed they could run further (or exercise more intensively) while listening to music. The latter four questions were only assessed in the conditions with attentional distraction. The first three questions were assessed after the tests with and without attentional distraction. 

### 2.4. Statistical Analyses

Data were analysed using SPSS software (version 21.0). Values of *p* < 0.05 were considered statistically significant. Effect of attentional distraction was studied using a 2 (condition: distraction *versus* no distraction) × 2 (group: normal-weight *versus* overweight) repeated measures analyses of variance. Results from items only obtained during the tests with distraction were analysed with independent samples *t*-tests.

Mediation of attentional distraction effects in primary outcomes (running distance and activity intensity) by attentional distraction effects in secondary outcomes (RPE, degree of annoyance, attention given to bodily sensations or thoughts about being able to carry on) was tested using a within-subject method suggested by Judd *et al.* [36]. In order to do this analysis, there must first be a distraction effect for both the primary outcomes (dependent variable) and secondary outcomes (mediator variables). Mediation analysis was performed by estimating a regression model where the difference in running distance/activity intensity (with music minus without music) was regressed onto the sum of the mediator variables and the difference of the mediator variables. If the regression coefficient for the difference predictor is significant, this indicates that differences in running distance/activity intensity are mediated by differences in the mediator variable. If the sum predictor, but not difference predictor, is mean-centered (each participant’s score subtracted from the mean score of the sample), complete mediation is indicated by a non-significant intercept [36]. In order to be able to assess complete mediation mean-centered sums of the mediator variables were included in the regression analyses.

## 3. Results

### 3.1. Descriptive Charateristics of Participants

Table 1 presents descriptive charateristics of the participants. There were no differences in age, height and leisure time index between overweight and normal-weight boys, but overweight boys had a higher weight and BMI and lower sport index compared to normal-weight peers.

**Table 1:** Descriptive characteristics of participants.

Characteristics	Normal-Weight (n = 33)	Overweight (n = 20)	*t*	*p*
age (yrs)	12.8 ± 0.6	12.8 ± 0.8	0.42	0.67
height (cm)	160.0 ± 9.4	163.2 ± 8.4	−1.63	0.11
weight (kg)	45.4 ± 7.3	70.3 ± 13.7	−7.5	<0.001
BMI (kg/m^2^)	17.9 ± 1.5	26.2 ± 3.7	−9.5	<0.001
leisure time index *	3.0 ± 0.6	3.1 ± 0.7	−0.52	0.61
sport index *	3.3 ± 0.7	2.9 ± 0.6	2.04	0.05

Note: * 5 point scale.

### 3.2. Field Running Performance

**Figure 1:**
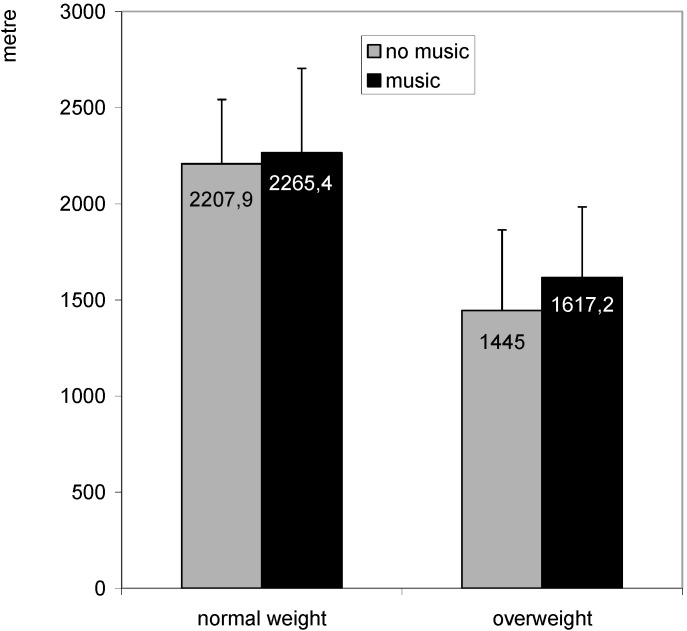
Running distance on the Cooper test with and without attentional distraction by music in normal-weight and overweight youngsters.

Primary outcomes: Running distances during the field tests are shown in Figure 1. There were no significant distraction by group interaction effects (F = 1.3, n.s.). Both overweight and normal-weight boys ran further in 12 min with music than without music (F = 5.0, *p* < 0.05). Overweight youngsters showed poorer performances compared to their normal-weight counterparts (F = 40.5, *p* < 0.001). 

Secondary outcomes: There were no significant distraction by group interaction effects for RPE (F = 0.6, n.s). With music (11.8 ± 2.9), participants reported lower RPE compared to without music (13.1 ± 3.7) (F = 5.9, *p* < 0.05). Overweight youngsters reported higher rates of perceived exertion after the running tests compared to their normal-weight counterparts (14.0 ± 3.5 *versus* 11.2 ± 2.8) (F = 11.9, *p* < 0.001). When asking how participants experienced the running tests, both overweight and normal-weight youngsters found the test less annoying with music (2.1 ± 1.1) compared to without music (2.8 ± 1.4) (F = 10.1, *p* < 0.01). They also reported to pay less attention to bodily sensations during the test with music (2.3 ± 1.0) compared to without music (2.7 ± 1.4) (F = 4.6, *p* < 0.05). Overall, degree of annoyance was higher in overweight (2.3 ± 1.1) compared to normal-weight youngsters (2.8 ± 1.5) (F = 5.3, *p* < 0.05). There were no significant differences between conditions or groups, nor interaction effects (F < 3.8, n.s.) regarding how often they had thoughts about being able to carry on with the test. Experiences during the test with music did not differ between overweight and normal-weight boys (*t* < 1.6, n.s). Both groups reported that they liked the self-selected music a lot (3.9 ± 1.4), that they could listen quite good to the music during the test (3.4 ± 1.3), that it was quite pleasant to run while listening to the music (3.3 ± 1.5) and that they believed they could run further while listening to music (3.6 ± 1.5). 

Mediation: As attentional distraction had a significant effect on (1) RPE, (2) how annoying they experienced the running test and (3) attention given to bodily sensations during the running test, mediation analyses were performed for these three potential mediators. Only the difference in degree of annoyance mediated the difference in running distance between both conditions (β = −0.46, *t* = −3.34, *p* < 0.01). The intercept of the regression was non-significant (*t* = 0.7, n.s.) indicating a complete mediation.

### 3.3. Activity during Exercise Session

**Figure 2:**
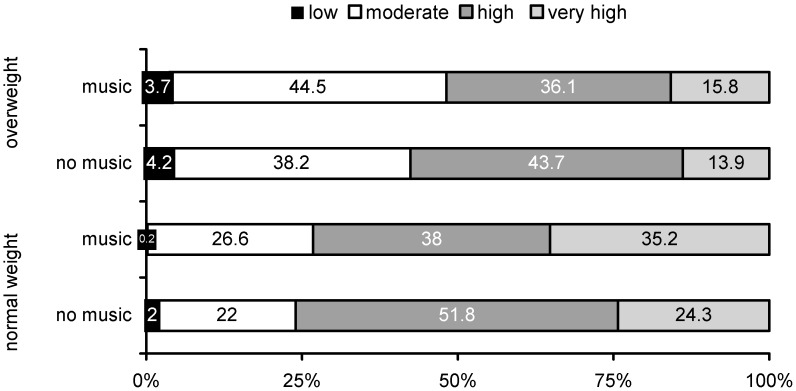
Proportion of time spent in activities of different intensities during the exercise session with and without attentional distraction by music in normal-weight and overweight participants.

Primary outcomes: In Figure 2 the total duration of the exercise session is equalled to 100%. The proportion of time spent in activities of different intensity is represented by the different coloured bars. There were no significant distraction by group interaction effects (F < 0.8, n.s). Both overweight and normal-weight boys exercised less at low and high intensity and more at moderate and very high intensity with music compared to without music (F = 6.0, *p* < 0.01). Overweight boys exercised a higher proportion of time at low and moderate intensity and a lower proportion of time at high and very high intensity, compared to the normal-weight boys (F = 4.0, *p* < 0.05). 

Secondary outcomes: In both overweight and normal-weight boys, RPE was lower in the condition with music (11.7 ± 3.0) than without music (12.9 ± 2.5) (F = 9.3, *p* < 0.01). Overweight boys reported higher RPE after the exercise session (13.0 ± 2.9) compared to their normal-weight peers (11.2 ± 3.3) (F = 5.9, *p* = 0.01). When asking how participants experienced the exercise sessions, both overweight and normal-weight youngsters found the session less annoying with music (2.5 ± 1.1) compared to without music (3.1 ± 1.5) (F = 7.1, *p* = 0.01). Both groups also reported to pay less attention to bodily sensations during the session with music (2.1 ± 1.1) compared to without music (2.4 ± 1.1) and to think less about being able to carry on with the exercise during the session with music (1.9 ± 1.2) compared to without music (2.2 ± 1.3), but these differences were not statistically significant (F = 2.1 and F = 3.2, n.s.). Although scores were generally somewhat higher in overweight compared to normal-weight boys, there were no significant group effects (F < 1.2, n.s.), nor distraction by group effects (F < 0.3, n.s.). Experiences during the exercise session with music did not differ between overweight and normal-weight participants (*t* < 1.9, n.s). Both groups reported that they quite liked the music (3.0 ± 1.4), that they could listen quite good to the music during the exercise session (2.9 ± 1.3), that it was quite pleasant to exercise while listening to the music (3.2 ± 1.5) and that they believed they could exercise more intensively while listening to music (3.4 ± 1.3). 

Mediation: As attentional distraction had a significant effect on (1) RPE and (2) how annoying they experienced the exercise session, mediation analyses were performed for these two potential mediators. Only the difference in rate of perceived exertion mediated the difference in mean activity intensity during the exercise session between both conditions (β = −0.37, *t* = −2.6, *p* = 0.01). The intercept of the regression was non-significant (*t* = 1.8, n.s.) indicating a complete mediation.

## 4. Discussion

The purpose of this study was to investigate the effect of attentional distraction by music on field running distances and on intensity of activity during an exercise session. We also wanted to investigate whether the effect of attentional distraction was moderated by overweight status and mediated by RPE, degree of annoyance, attention given to bodily sensations or thoughts about being able to carry on.

Results clearly indicated that both overweight and normal-weight boys ran further in 12 min while listening to their favourite piece of music compared to without music. This finding is in line with previous studies in students and athletes [17] and with the previous laboratory experiment in obese youngsters [18]. Further, we also found that both overweight and normal-weight youngsters were exercising at a higher intensity during the exercise session with music compared to without music. With music, some proportion of time spent in low intensity activity was probably replaced by moderate intensity activity and some proportion of time spent in high intensity activity was replaced by very high intensity activity. To our knowledge, this is the first study to investigate the effect of attentional distraction within the context of an exercise session. Since participants were exercising at a higher intensity while listening to music, more energy was expended, which is the main aim of any exercise program for overweight youngsters. 

Previous studies suggest different ways in which music may enhance exercise performance. Szmedra and Bacharach [37] suggest that music might allow participants to relax and reduce muscle tension, thereby increasing blood flow and lactate clearance while decreasing lactate production in the working muscles and consequently having a psychobiological impact on exercise. Further, it is assumed that the exerciser has a limited attentional capacity [38,39]. During exercise individuals have access to internal sensory information (such as heart rate, breathing, pain,…) and external environmental cues (such as noise, music, other exercisers, scenery) that compete for attentional focus [40]. So, turning participants’ attention away from internal cues resulting from physiological stimuli through some distracter (such as music) during exercise, will prevent them from focusing on feelings of discomfort associated with exercise and will reduce perceived exertion. This hypothesis has been confirmed by several previous studies in adults [37,41,42,43,44,45,46,47,48,49] and is also in agreement with the findings of this study. With music, participants reported lower RPE and paid less attention to bodily sensations compared to without music. However, only RPE and not attention paid to bodily sensations was found to be a mediator of the effect of attentional distraction and a decrease in RPE only mediated the effect of attentional distraction on activity intensity during the exercise session, but not on field running performance. Some studies also suggest that music enhances enjoyment levels during exercise [40,43,49,50]. Music may influence emotions and mask unpleasant feelings during exercise [51]. In this study, participants found the running test and the exercise session with music less annoying compared to without music. They may associate the music with positive past experiences, they may indulge in pleasant fantasizing or may focus attention on pleasant future events which may improve emotional or affective state during exercise [51]. Synchronisation of music with exercise may have a psyching-up effect [41]. However, a decrease in feelings of annoyance only mediated the effect of attentional distraction on field running performance, but not on activity intensity during the exercise session. From the mediation analyses, we can conclude that the effect of attentional distraction works through different mechanisms depending on the type of activity. Effect of music on the running test, which is a very monotonous and less pleasant activity, is mediated by feelings of annoyance, while the effect of music on activity intensity during the exercise session, which is a more diverse and pleasant activity, is mediated by RPE.

Previous research showed that the effect of attentional distraction is different in trained *versus* untrained athletes [52]. Elite and novice athletes employ different cognitive coping strategies to meet exercise demands [53]. Trained athletes direct their focus to internal cues during exercise in order to adapt pace and intensity to the functional information of bodily sensations. Brownley *et al.* [54] demonstrated that listening to fast, upbeat music during exercise is beneficial for untrained runners but counterproductive for trained runners. We hypothesized that the effect of attentional distraction would also be different in overweight compared to normal-weight youngsters. Overweight youngsters report more physical complaints while exercising [8]. Therefore we expected the effect of attentional distraction to be stronger in overweight compared to normal-weight youngsters. However, this hypothesis was not confirmed, the effect of attentional distraction was similar in both groups. 

Although this was not the main purpose of this study, we also found that overweight youngsters showed poorer performances on the field running tests. This is in agreement with findings of previous studies [21,55,56]. This poorer performance in this weight-bearing activity in overweight youngsters is probably mainly due to the fact that excess body fat adds to the mass of the body without contributing to its force producing capability, thus becoming an inert load to be moved during running. Another explanation could be that overweight youngsters avoid running because of the greater energy cost required to move the total body. In this case the poorer performance could be the consequence of a lack of experience in running. Overweight youngsters also exercised at a lower activity intensity during the exercise sessions compared to normal-weight counterparts. Although performances were generally lower in overweight youngsters, they reported higher rates of perceived exertion and they found the exercise session more annoying compared to normal-weight youngsters. Obese children generally rate perceived exertion higher than normal-weight counterparts when subjected to standardised workload on a treadmill [57,58]. These higher rates of perceived exertion and annoyance are in line with previous findings that overweight youngsters report more physical complaints and perceive less enjoyment while exercising [8]. Since this study demonstrated that attentional distraction by music increases exercise performance and intensity without increasing physical complaints or annoyance, this might be a useful strategy to increase enjoyment and exercise adherence in overweight youngsters. Future research needs to investigate whether listening to music also has a positive effect on motivation to exercise. As exercising while listening to music is perceived as less annoying or more pleasant, this might increase intrinsic motivation [59].

This was the first study to investigate the effect of attentional distraction in overweight and normal-weight youngsters in field settings. Previous studies in this field were conducted in normal-weight adults, only one laboratory study was conducted in obese youngsters [18]. However, this study has some limitations. First, the results of this study are limited to overweight boys and cannot be generalised to overweight girls. Secondly, it is possible that there were differences in sexual or skeletal maturity between normal-weight and overweight boys. Unfortunately we were not able to assess this. Thirdly, it is unknown which features of the music were critical in obtaining the distraction effect. Previous studies showed that the effect of distraction may depend on type of music, music tempo, music loudness, synchronisation with exercise or emotional significance of the music [17]. Next, the effect of distraction on exercise intensity was limited to a standardised 20 min exercise session. Further research is needed to investigate whether attentional distraction also works within a more comprehensive exercise program consisting of a variety of activities. Finally, in this study attention was distracted by music, the usefulness of other forms of distractions such as watching a video or environmental distraction (f.i. running on the beach or in a forest) needs further investigation. Our experience in overweight 6 to 12 year old children is that making activities part of an exciting adventure works well as a way of distraction. 

## 5. Conclusions

The present study showed that attentional distraction by music has a positive effect on running distances on a field endurance test and on activity intensity during an exercise session. The effect on the field endurance test was mediated by feelings of annoyance, while the effect on activity intensity during the exercise session was mediated by RPE. This indicates that the effect of attentional distraction is working through different mechanisms depending on the type of activity. Despite our hypothesis that the effect of attentional distraction would be stronger in overweight compared to normal-weight youngsters, the effect was similar in both groups. Motivating overweight youngsters to exercise at high enough intensity is a big challenge. Music may help overweight adolescents to enjoy physical activity and adhere to higher intensity physical activity. Further research is needed to investigate whether attentional distraction is a useful technique to increase exercise adoption and adherence in obesity prevention and treatment. 
